# Treatment success with two doses of methotrexate vs single dose of methotrexate in Ectopic Tubal Pregnancy

**DOI:** 10.12669/pjms.38.6.5251

**Published:** 2022

**Authors:** Mehnaz Khakwani, Rashida Parveen, Syeda Ali

**Affiliations:** 1 Mehnaz Khakwani, FCPS (Obs & Gyne). Department of Obstetrics and Gyne, Unit-1, Nishtar Medical University, Multan, Pakistan; 2 Rashida Parveen, FCPS (Obs & Gyne). Department of Obstetrics and Gyne, Unit-1, Nishtar Medical University, Multan, Pakistan; 3 Syeda Ali, FCPS (Obs & Gyne). Department of Obstetrics and Gyne, Unit-1, Nishtar Medical University, Multan, Pakistan

**Keywords:** Beta-human chorionic gonadotrophin, Ectopic pregnancy, Methotrexate

## Abstract

**Objectives:**

To compare the success rates and safety of two-doses of methotrexate versus single dose of methotrexate in ectopic tubal pregnancy.

**Methods:**

This was an open-label, randomized controlled trial done at “The Department of Obstetrics & Gynecology, Nishtar University Hospital, Multan” from January 2020 to July 2021. A total of 100 women (50 in each group), aged 20 to 35 years with a tubal ectopic pregnancy were enrolled. All patients were randomly allocated to either single-dose or two-dose methotrexate protocol. Cases were evaluated for treatment success, side effects, beta-human chorionic gonadotrophin (β-hCG) resolution time and treatment satisfaction.

**Results:**

In a total of 100 cases, mean age was 29.6±4.5 years. Mean serum β -hCG levels at baseline was 1212±78 mIU/ml. Treatment success was noted among 43 (86.0%) cases of single-dose group versus 45 (90.0%) cases (p=0.5382). Duration of β -hCG resolution time was significantly shorter in two-dose group (23.0±12.1 days versus 28.2±12.8 days, p=0.0394). No significant difference was noted in methotrexate associated side effects in both study groups (p=0.9996). Overall, mean satisfaction score was 4.0±1.3 (out of 5).

**Conclusion:**

Although, β -hCG resolution time was significantly low in two-dose protocol but single-dose methotrexate offered comparable success rates versus two-dose protocol. Side effects were mild and comparable in both methotrexate treatment protocols. Methotrexate was found to be effective in the medical management of ectopic pregnancy.

***Trial Registry:*** NCT05208034 at: www.ClinicalTrials.gov

## INTRODUCTION

Ectopic pregnancy (EP) is estimated to be responsible for approximately 20% of all pregnancy-related mortality and 46% early pregnancy mortality.^1^ Hemodynamically stable women with EP are frequently managed with methotrexate (MTX) while multiple protocols like fixed multiple doses, single-dose as well as two-dose regimens have been in practice for treating EP, but no consensus exists regarding the optimum dosage regimen.^2^-^4^ Literature reports multiple dosage regimens of MTX to be associated with increased rates of side effects. Single dose protocol has good compliance and fewer side effects but is linked with lower success rates in comparison to multiple dose protocols.^5^-^7^

A new treatment protocol involving “two-doses” of MTX for medical management of EP was introduced in 2007 but most of the research conducted so far has been retrospective in nature and limitations in study designs.^8^-^10^ No such study in recent years has been done in Pakistan to compare the success and safety of single-dose and two-dose MTX protocols so this study was planned to compare the success rates and safety of two-doses of MTX versus single dose of MTX in tubal EP.

## METHODS

This open-label, randomized controlled trial was done at “The Department of Obstetrics & Gynecology, Nishtar University Hospital, Multan Pakistan” from January 2020 to July 2021. With 95% confidence and 80% power, considering success rates of two-dose versus single-dose protocols of MTX in patient with EP as 64.7% and 90.2% respectively, a minimum sample of size of 92 patients (46 in each group) including 10% dropout was calculated.^9^,^11^

Inclusion criteria was hemodynamically stable women aged 20-35 years with diagnosis of tubal EP having pre-treatment beta-human chorionic gonadotrophin (β-hCG) level below 1500 mIU/ml, gestational sac with largest diameter as 4cm and willing to take MTX treatment and follow up. Tubal EP was diagnosed adopting transvaginal ultrasound approach along with medical history evaluation, physical examination, transvaginal ultrasonography and measurement of β-hCG level. Exclusion criteria was women with heterotrophic pregnancy or persistent tubal pregnancy, or embryonic cardiac motion, suspected tubal rupture or those with harmful effects of MTX treatment on organ functions.

**Fig.1:**
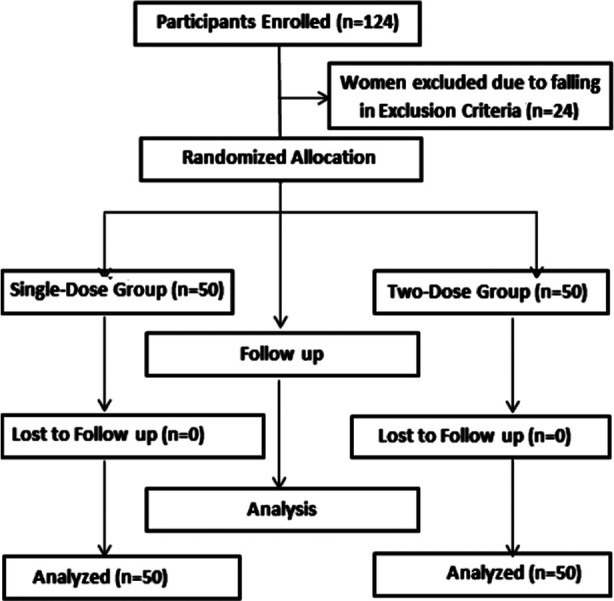
Methodology flow chart.

Approval for this study and its treatment regimens were acquired from “Institutional Review Board” (Ref#15501, dated: 7-8-2021). Initially, a total of 124 patients were considered but 24 patients were excluded due to falling in exclusion criteria so finally, 100 patients (50 in each group) were enrolled for this study (Fig.1). Participants were randomly allocated to both groups as 1:1 ratio. Randomization was done through computer generated numbers to either single-dose or two-dose group. Women in single-dose group were administered a single-dose of intramuscular MTX as 50 mg/m^2^ on day-zero (the start of treatment). Measurement of β-hCG levels was done at day-4 and 7 and if β-hCG levels fell below 15 IU between day-4 and day-7, the treatment was labeled successful. Women allocated to two-dose group were administered intramuscular MTX as 50mg/m^2^ at day-zero and 7 while measurement of β-hCG was ordered at day-14. In case if β-hCG level fell below 15 IU at day-14 in two-dose group, the treatment was labeled as success. At any point of time, cases were sent for surgical treatment if suspected for EP rupture or if treating physician advised for any cause. All women undergoing surgical treatment were labeled as failure of medical treatment. All women were followed up weekly for evaluation of β-hCG levels until the time when β-hCG levels fell below 5 mIU/ml and duration was noted. All cases were evaluated for success or failure of the treatment protocol in both groups.

Along with success of treatment, duration of β-hCG resolution time below 5mIU/ml, MTX-associated side effects and treatment satisfaction (likert scale scores as 1,2,3,4 or 5 showing very dissatisfied, dissatisfied, not sure, satisfied or very satisfied respectively) were noted. All women were asked whether they would elect for the same treatment protocol again if needed in the future. A special proforma was formed to record all study information.

Data analysis was performed using SPSS version 26.0. For quantitative data, mean and standard deviation (SD) were calculated while frequencies and percentages were highlighted for qualitative variables. The student t-test was employed for comparing means while chi-square was utilized for qualitative variables taking p<0.05 as significant.

## RESULTS

In a total of 100 cases, mean age was noted to be 29.6±4.5 years. There were 58 (58.0%) women who were nulliparous. Previous history of abortion and EP were noted in 9 (9.0%) and 5 (5.0%) cases respectively. Overall, mean duration of gestation was noted to be 45.4±14.1 days. Mean serum β-hCG levels at baseline was 1212±78 mIU/ml. Table-I is showing comparison of baseline characteristics of cases in both study groups.

**Table I:** Baseline Characteristics (N=100).

Baseline Characteristics		Sing-Dose Group (n=50)	Two-Dose Group (n=50)	P-Value
Age in Years (Mean±SD)		29.4±4.3	30.1±4.6	0.4337
BMI in kg/m^2^ (Mean±SD)		21.4±3.1	21.8±2.9	0.5068
Parity Status	Nulliparous	30 (60.0%)	28 (56.0%)	0.6853
	Multiparous	20 (40.0%)	22 (44.0%)	
Previous History of Abortion		4 (8.0%)	5 (10.0%)	0.7177
Previous History of Ectopic Pregnancy		3 (6.0%)	2 (4.0%)	0.6464
Duration of Gestations in Days (Mean±SD)		44.7±13.9	46.0±14.5	0.6482
Serum β-hCG Level in mIU/ml (Mean±SD)		1202±68	1221±75	0.1876
Maximal Diameter of Ectopic Mass in cm (Mean±SD)		2.63±0.61	2.66±0.64	0.8109

Overall, treatment success was noted in 88 (88.0%) cases. In terms of primary outcome, treatment success was noted among 43 (86.0%) cases of single-dose group versus 45 (90.0%) cases (p=0.5382). Duration of β-hCG resolution was significantly shorter in two-dose group in comparison to single-dose group (23.0±12.1 days versus 28.2±12.8 days, p=0.0394). Abdominal pain was the commonest side effect reported in 14 (14.0%) cases while there was no significant difference in MTX-associated side effects was noted among cases of both study groups (p=0.9996). There were 79 (79.0%) cases among both study groups who were willing to undergo same treatment protocols in case EP occur in the future as well (p=0.4614). Overall, mean satisfaction score was 4.0±1.3 while it was noted to be 3.8±1.3 and 4.1±1.4 in single-dose and two-dose group (p=0.2696).

**Table II:** Treatment Outcome, Safety and Patient Satisfaction in Both Groups (N=100).

Treatment Outcome		Single-Dose Group (n=50)	Two-Dose Group (n=50)	P-Value
Treatment Success		43 (86.0%)	45 (90.0%)	0.5382
β-hCG Resolution Time in Days (Mean±SD)		28.2±12.8	23.0±12.1	0.0394
Frequency of Side Effects	Abdominal Pain	6 (12.0%)	8 (8.0%)	0.9996
	Nausea and/or Vomiting	3 (6.0%)	4 (8.0%)	
	Sore Throat	3 (6.0%)	4 (8.0%)	
	Elevated Alanine Aminotransferase	2 (4.0%)	3 (6.0%)	
	Leukopenia and/or Thrombocytopenia	1 (2.0%)	1 (2.0%)	
Treatment Satisfaction Score (Mean±SD)		3.8±1.3	4.1±1.4	0.2696
Willingness to Undergo Same Treatment Protocol If Ectopic Pregnancy Occurs in the Future		41 (82.0%)	38 (76.0%)	0.4614

## DISCUSSION

The EP is known to be one of the most important causes of maternal morbidity as well as mortality while late diagnosis of EP leading to rupture and internal hemorrhage is a fearsome complication. In unruptured EP, MTX is considered as an attractive medical treatment option but controversies exist regarding the best medical management protocols.

In the present study, mean age was noted to be 29.6±±4.5 years among study cases. This was very similar to a study conducted in Iran^12^ where the authors in a retrospective analysis of 370 women with tubal EP found mean age to be 29.3±5.6 years. A local study from Islamabad^13^ evaluating 52 women with EP found mean age to be 28±4.8 years which is very close to what we noted.

Lots of literature reports utilization of MTX as medical management of EP while overall effectiveness of MTX has been reported to be ranging between 65-97%.^14^-^17^ In the present study, overall success of MTX was 88% which shows its effectiveness. The two-dose treatment protocol of MTX was put forward by Barnhart and colleagues in 2007^16^ and it was exhibited that this treatment regimen minimized the number of MTX injections and follow up visits in comparison to those cases who received fixed multi-dose protocol. Barnhart and colleagues shared the success rates of two-dose regimen of MTX to be 87% while no major side-effects were reported.^16^ Barnhart et al.^16^ found two-dose protocol to not exhibit significantly superior success rates when compared to single-dose protocol. Co-relating with the findings of Barnhart et al, we noted treatment success rates of single-dose versus two-dose protocol to be 86.0% and 90.0% respectively while no statistically significant difference was reported in our findings (p=0.5382). Hamed et al. in 2012^18^ comparing single-dose versus two-dose treatment protocol of MTX for treating women with EP revealed single-dose of MTX to show success rate of 82% versus 89% in two-dose protocol group while the difference was not statistically significant which shows similarity with the current research. Kanmaz et al.^17^ found success rate of single-dose protocol of MTX to be 87% in comparison to 90% in two-dose protocol but both treatment protocols were found to be comparable with each other. Some researchers^19^ have shared that success rates of among women with EP and higher β –hCG levels (>5000 mIU/ml) with single-dose of MTX are low in comparison to two-doses of MTX (59% versus 80%) but as we only included women but as we only included women who had β –hCG levels below 1500 mIU/ml which could be the reason that we did not observe such kind of a difference in our cases. Likewise, a recent meta-analysis concluded that women having EP and high levels of β –hCG respond significantly better with two-doses of MTX when compared to a single-dose.^20^

Present study is 1^st^ of its kind from Pakistan where we had planned to find out success rates of single-dose versus two-doses of MTX along with observation of most common side effects and treatment satisfaction among women with tubal EP.

### Limitations of the study

As this was a single center study with a relatively small sample size, our findings cannot be generalized. Randomized clinical trials involving multiple settings and larger sample size can further shed light on the findings of this research.

## CONCLUSION

Although, β -hCG resolution time was significantly low in two-dose protocol but single-dose methotrexate offered comparable success rates versus two-dose protocol. Side effects were mild and comparable in both methotrexate treatment protocols. Methotrexate was found to be effective in the medical management of ectopic pregnancy.

### Authors’ Contribution:

**MK:** Conceived, Responsible for data’s integrity and authenticity.

**RP:** Data Collection, Data Analysis, Drafting.

**SA:** Literature Review, Discussion.
